# Measuring the dynamic structure factor of a quantum gas undergoing a structural phase transition

**DOI:** 10.1038/ncomms8046

**Published:** 2015-05-06

**Authors:** Renate Landig, Ferdinand Brennecke, Rafael Mottl, Tobias Donner, Tilman Esslinger

**Affiliations:** 1 Institute for Quantum Electronics, ETH Zürich, CH—8093 Zürich, Switzerland; 2 Physikalisches Institut, University of Bonn, Wegelerstrasse 8, 53115 Bonn, Germany

## Abstract

The dynamic structure factor is a central quantity describing the physics of quantum many-body systems, capturing structure and collective excitations of a material. In condensed matter, it can be measured via inelastic neutron scattering, which is an energy-resolving probe for the density fluctuations. In ultracold atoms, a similar approach could so far not be applied because of the diluteness of the system. Here we report on a direct, real-time and nondestructive measurement of the dynamic structure factor of a quantum gas exhibiting cavity-mediated long-range interactions. The technique relies on inelastic scattering of photons, stimulated by the enhanced vacuum field inside a high finesse optical cavity. We extract the density fluctuations, their energy and lifetime while the system undergoes a structural phase transition. We observe an occupation of the relevant quasi-particle mode on the level of a few excitations, and provide a theoretical description of this dissipative quantum many-body system.

## Abstract (web summary)

The dynamic structure factor is measured in condensed matter systems via neutron scattering, but this is not applicable to ultracold atomic gases. Here, the authors show how it can be measured relying on the strongly enhanced inelastic scattering of photons off a quantum gas in an optical cavity.

## Graphical abstract

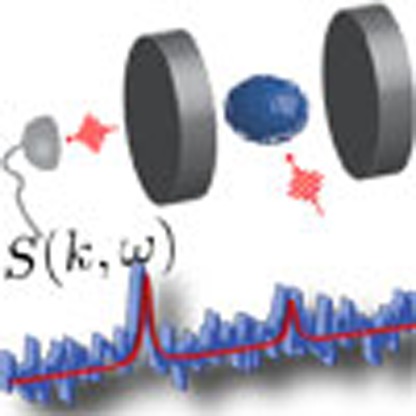

An interacting quantum many-body system can be characterized by analysing its response to a weak perturbation. In the framework of linear response theory, a key quantity is the dynamic structure factor, which is the Fourier transform of the spatial and temporal density–density correlations^1^,^2^. Knowledge of the dynamic structure factor provides a complete picture of the emerging quasi-particle modes^3^, their excitation energy, lifetime and mean occupation number. These quasi-particle modes determine the collective density fluctuations of the system and may also characterize the critical behaviour in the vicinity of a phase transition^4^. For example, in a long-range interacting system, a structural phase transition can be driven by a roton-like mode softening^5^,^6^,^7^,^8^, which is expected to show up as a thermally enhanced peak in the dynamic structure factor^9^.

In solid state systems, the dynamic structure factor *S*(**k**,*ω*) can be measured by illuminating a sample with a beam of neutrons^10^ or X-rays^11^, and analysing the inelastically scattered particles with regard to their change in energy *ℏω* and momentum *ℏ***k**. In dilute quantum gases, a measurable signal has only been obtained from photons elastically scattered off a density-modulated sample^12^,^13^,^14^,^15^. Direct detection of inelastically scattered photons into free space^16^, in analogy to neutron scattering, is however hindered by a vanishingly small signal^17^, see Fig. 1a.

A technique measuring the spectral response function of a quantum gas, that is, the dynamic structure factor at zero temperature^18^, is Bragg spectroscopy^19^,^20^. It is based on stimulated rather than spontaneous inelastic scattering of photons between two laser beams. Therefore, the transfer of momentum and energy to the atomic cloud is predetermined by the angle and frequency difference between the beams, and is typically measured via destructive absorption imaging. A complementary detection method analyses the change in light field intensity in one of the two Bragg beams^21^ and could in principle be extended with the help of cavities to be only weakly perturbative^22^. However, all these methods measure the linear response of the gas on a perturbation and are insensitive to thermally excited quasi-particles. A different approach, *in situ* imaging, has been used to extract the temperature-dependent static structure factor *S*(**k**)=∫*dωS*(**k**,*ω*) of a two-dimensional gas^23^. Similar to the analysis of noise correlations from images of ballistically expanded ultracold gases^24^, this approach gives no access to the quasi-particle spectrum, that is, the temporal dynamics.

Here we present a nondestructive, direct measurement of the dynamic structure factor at distinct **k**-vectors in a Bose–Einstein condensate (BEC) undergoing a structural phase transition induced by cavity-mediated long-range interactions. We place a BEC into an ultrahigh finesse optical cavity^25^,^26^ and illuminate the atoms with a transverse laser field. The enhanced vacuum field inside the optical resonator^27^ increases the spontaneous inelastic scattering of photons into the cavity mode by several orders of magnitude, so that photons leaking out of the cavity mode give rise to a detectable signal and access to the density correlations in real time, see Fig. 1b. Therefore, density fluctuations in the gas are mapped onto fluctuations of the light field, which then can be directly accessed.

## Results

### System description

In our experiment, the transverse laser field acts simultaneously as a pump field controlling the long-range interactions^8^,^28^. This field has a frequency *ω*_p_ and a wavevector **k**_p_, and is in a standing-wave configuration directed perpendicularly to the cavity mode. It is far detuned from atomic resonance to avoid electronic excitation of the atoms. At the same time, it is detuned by only a few cavity linewidths from the cavity resonance, which enables vacuum-stimulated scattering of pump photons into the cavity mode at wavevector **k**_c_. These two-photon processes mediate long-range atom–atom interactions in the BEC, giving rise to a roton-like mode softening^8^ and a structural phase transition^28^,^29^. The same two-photon processes are exploited for detection. The light scattered from the transverse pump field into the cavity mode can be regarded as a superposition of all field amplitudes scattered by the individual atoms. It thus carries information on the density–density correlations of the gas at the wavevectors **k**_cb_=±**k**_p_±**k**_c_, which are determined by the underlying two-photon processes^29^. Within the cavity linewidth, which is two orders of magnitude larger than the frequency of the relevant quasi-particle excitation, the energies of the photons stimulated into the vacuum mode are not fixed. This is in contrast to Bragg spectroscopy, where the energy of the quasi-particle excitations that are created during probing is determined by the frequency difference of the classical fields driving the two-photon processes. The spectral analysis of the light field leaking out of the cavity thus gives us direct access to the dynamic structure factor at finite temperatures.

As previously described^8^,^28^,^29^,^30^, we trap a BEC of *N*=1.0(1) × 10^5 87^Rb atoms at the centre of an ultrahigh-finesse optical Fabry–Pérot cavity and illuminate it by the transverse pump field. The cavity-mediated interaction leads to the formation of a quasi-particle mode, which is a superposition of the collective momentum excitation at wavevectors **k**_cb_ of the BEC and a tiny admixture of photons inside the cavity (see Supplementary Note 1). Neglecting atom–atom collisions, its energy *ℏω*_s_ equals in the limit of zero pump power *P* the bare energy 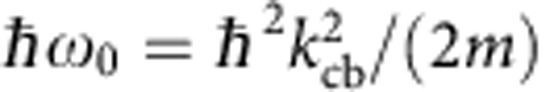 of a single momentum excitation and decreases with increasing power^8^, where *m* denotes the atomic mass. Owing to this mode softening, the energy *ℏω*_s_ of the quasi-particle mode approaches zero at a critical pump power *P*_cr_, which leads to a phase transition from a normal state with a flat density distribution to a self-organized state with checkerboard density modulation. The emergent density structure leads to elastic scattering of transverse pump light and a macroscopic population of the cavity mode. An order parameter of this phase transition is the expectation value of the operator 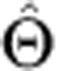 describing the overlap of the atomic density 
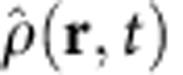
 and the checkerboard mode structure, 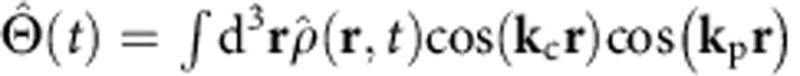.

### Dynamic structure factor

As the cavity decay rate *κ*=2*π* × 1.25 MHz is more than two orders of magnitude faster than the evolution rate *ω*_s_ of the coupled system, the light field 
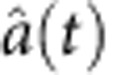
 inside the cavity adiabatically follows the order parameter, 
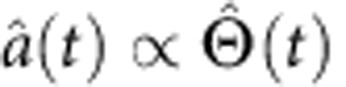
 (ref. 29). The frequency spectrum of the light leaking out of the cavity thus reveals the temporal and spatial Fourier transform of the atomic density correlations, evaluated at one particular wavevector. Specifically, the dynamic structure factor of the system at wavevector **k**_cb_ is related to the cavity field according to


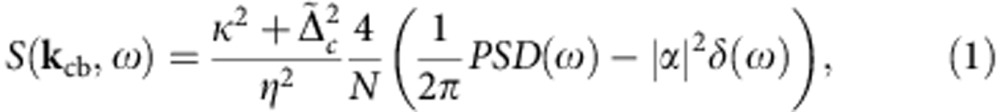


where *ω* is the frequency shift of the cavity output field from the pump light frequency *ω*_p_ because of inelastic scattering. *PSD*(*ω*) is the power spectral density of the intracavity light field with the mean coherent field amplitude 
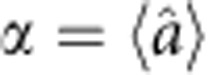
, and *η* is the two-photon Rabi-frequency of the scattering process, proportional to 
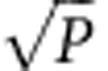
. 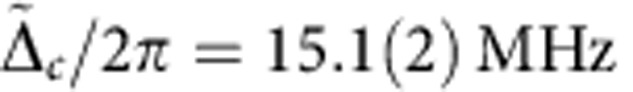 is the detuning between the pump laser frequency and the dispersively shifted cavity resonance (see Supplementary Note 1).

**Figure 2:**
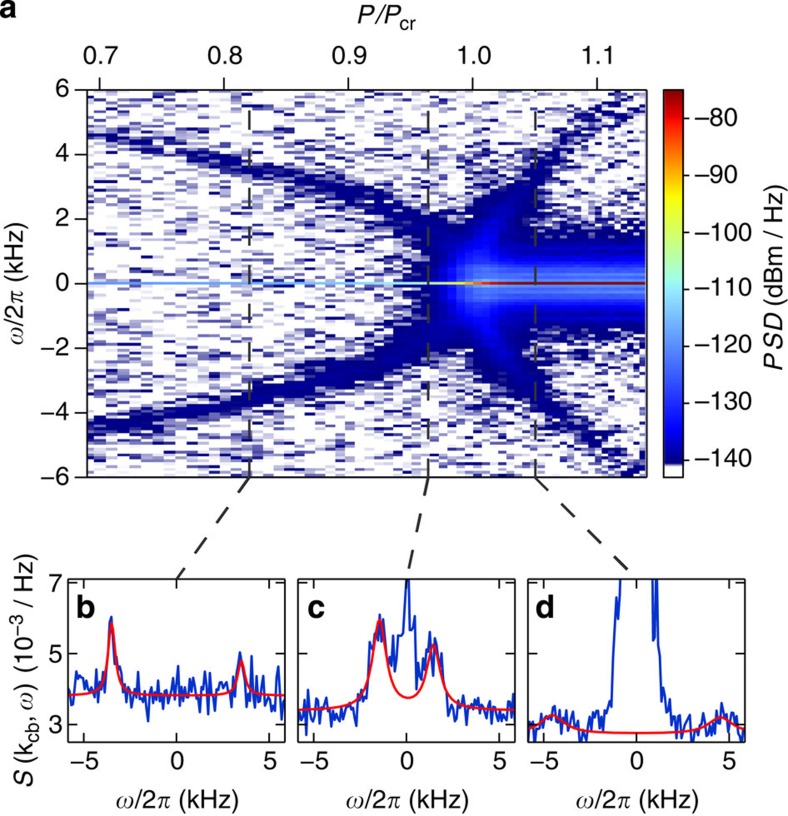
Power spectral density and dynamic structure factor. (**a**) The power spectral density *PSD* of the light field leaking out of the cavity is shown as a function of frequency shift *ω* with respect to *ω*_p_ and relative transverse pump power *P*/*P*_cr_ (average over 147 experimental repetitions). Two sidebands are visible, corresponding to the incoherent creation (*ω*<0) and annihilation (*ω*>0) of quasi-particles. The energy of these quasi-particles vanishes towards the critical point. At the phase transition, a strong coherent field at the pump frequency appears (*ω*=0). We attribute the broadened feature around *ω*=0 to residual low-frequency technical noise in our system. Note the large dynamic range of the data on the logarithmic scale. The panels (**b**–**d**) show the normalized dynamic structure factor S(**k**_cb_, *ω*) for three different values of *P*/*P*_cr_ (see dashed lines in upper panel), derived from the power spectral density *PSD*(*ω*). While the position and width of the sidebands give direct access to the energy and lifetime of the quasi-particles, the sideband asymmetry can be used to determine the occupation of the quasi-particle mode. Red line shows a fit to the sidebands with our theoretical model to extract these properties.

To analyse the light field leaking out of the cavity, we use a balanced heterodyne detection scheme^30^. Figure 2 shows the power spectral density *PSD*(*ω*) of the light field as we linearly increase the transverse pump power *P* across the critical point. The rate of change of *P*/*P*_cr_ is a few Hertz, such that the system can be assumed to adiabatically follow its steady state throughout the measurement^29^,^30^. The small panels in Fig. 2 show examples of *S*(**k**_cb_,*ω*) for different values of *P*/*P*_cr_, converted via equation (1) and normalized to unity for the noninteracting case^31^. The data reflect the microscopic processes taking place: pump photons of frequency *ω*_p_ inelastically scattered at the atomic ensemble will be shifted in their frequency. They become visible as red (blue) sideband at frequency *ω*_p_−*ω*_s_ (*ω*_p_+*ω*_s_) if they create (annihilate) a quasi-particle. We observe the corresponding sidebands whose frequency shift tends to zero when approaching the critical point at *P*/*P*_cr_=1 from either side of the phase transition. These density fluctuations can be distinguished from a checkerboard density modulation at **k**_cb_ at which pump photons will be elastically scattered without a frequency shift, visible at *ω*=0. Intuitively, this light field arises from scattering at Bragg planes in the density-modulated cloud. The transverse pump power *P* influences not only the effective long-range interactions in the system but also the measurement process itself. For increasing pump power, the measurement imprecision due to the shot noise of the transverse pump field becomes less relevant, as can be seen from the decreasing background level of *S*(**k**_cb_,*ω*)^32^. The influence of the inevitable measurement backaction will be discussed further below.

**Figure 3:**
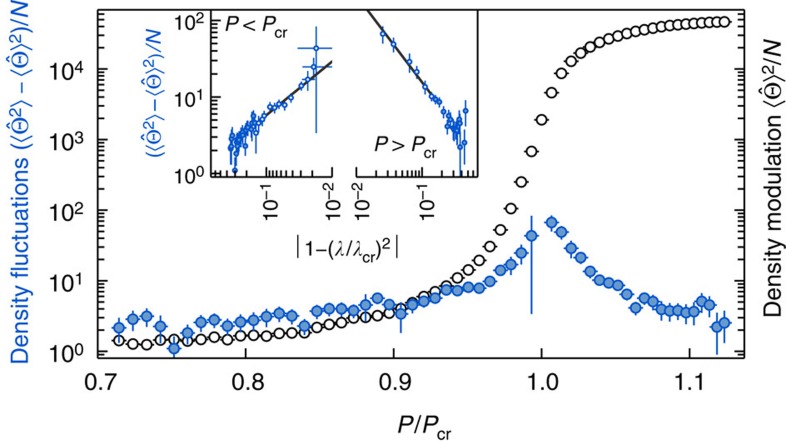
Density fluctuations and density modulation. The variance of density fluctuations (filled blue symbols) and the square of the density modulation (open black symbols) of the long-range interacting quantum gas is shown as a function of relative pump power *P*/*P*_cr_. The fluctuation data are extracted from the dynamic structure factor (see Fig. 2) by integrating over the fit function describing the sidebands and is proportional to the static structure factor. The coherent density modulation is calculated from the power spectral density at the zero frequency bin. The vertical error bars display the statistical error (s. d.) from the fit, while the horizontal error bars display the s.d. in our determination of the critical point. The inset displays a double logarithmic plot to demonstrate the scaling behaviour of the variance of the density fluctuations against the distance to the critical point, expressed as the Hamiltonian coupling parameter *λ* (see Supplementary Note 1). From a linear fit, we find critical exponents of 0.7(1) and 1.1(1) on the normal and self-organized side, respectively. The open symbols in the inset are used for the fitting.

The total power of elastically scattered light is proportional to the square of the density modulation 
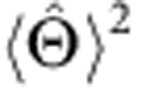
 and is displayed as open symbols in Fig. 3. This coherent density modulation increases over five orders of magnitude while crossing the critical point. In the normal phase (*P*/*P*_cr_<1), we also observe a weak field at the pump laser frequency (that is, at *ω*=0), which originates from a small symmetry-breaking field caused by the finite size of the system and residual scattering of the transverse pump beam at the cavity mirrors^29^,^30^.

The total power of frequency-shifted light is proportional to the variance of the checkerboard density fluctuation 
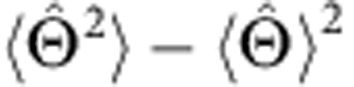
, and thus to the static structure factor *S*(**k**_cb_)=∫d*ω S*(**k**_cb_,*ω*). We show the variance of density fluctuations as filled symbols in Fig. 3 and observe a divergence when approaching the critical point *P*/*P*_cr_=1 from either side, heralding a second-order phase transition. The inset displays the variance of the density fluctuations on a double logarithmic plot to illustrate the scaling behaviour. The variance is plotted as a function of the Hamiltonian coupling parameter *λ*, derived from measured quantities and using a theoretical model (see Supplementary Note 1). In the normal and the self-organized phase, we extract critical exponents of 0.7(1) and 1.1(1), respectively. We attribute the deviation from our previous measurement in the normal phase, which gave 0.9(1) (ref. 29), to the refined model used for the scaling of the horizontal axis and the improved measurement scheme that allows us to directly distinguish between density fluctuations and a density modulation. Current theoretical research taking into account the open character of the system due to cavity dissipation predicts an exponent of 1.0 (refs 33, 34). Also the influence of thermal noise was theoretically shown to lead to an exponent of 1.0 (refs 35, 36). The difference between the experimentally observed exponent and the predicted value might originate from finite size effects and the presence of a small symmetry-breaking field. Further, the theory models do not include damping of the momentum excitation because of atom–atom collisions^37^,^38^,^39^.

### Characterization of the quasi-particle mode

**Figure 4:**
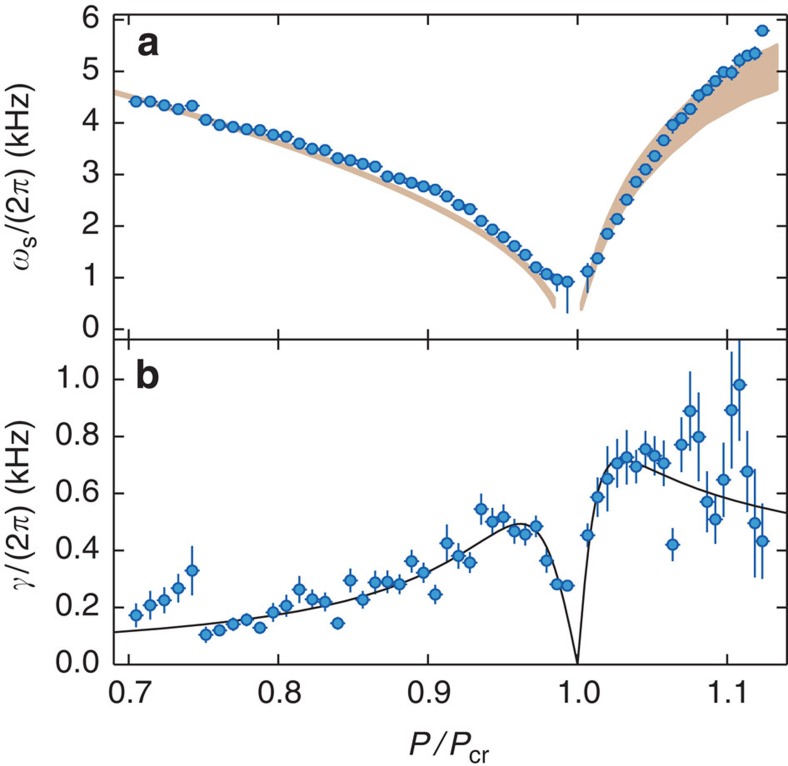
Characterization of the quasi-particle mode. Frequency *ω*_s_ (**a**) and decay rate *γ* (**b**) of the quasi-particles as a function of relative pump power *P*/*P*_cr_, as extracted from the fit to the dynamic structure factor (Fig. 2). The grey-shaded area in the top panel results from an *ab initio* calculation of the expected soft mode frequency, taking into account the experimental uncertainties in the determination of the coherent cavity field and the depth of the optical lattice resulting from the transverse pump field. Close to *P*/*P*_cr_=1, for *ω*_s_/(2*π*)<400 Hz, the uncertainty in modelling atom loss leads to a substructure, which we omit in the graph. The solid line in the lower panel is a fit with a phenomenological function to the data (see Supplementary Note 3). Vertical and horizontal error bars indicate the statistical errors (s. d.) reported from the fit, and the error (s.d.) in the determination of the critical point, respectively.

The access to the dynamic structure factor *S*(**k**_cb_,*ω*) allows us to characterize the quasi-particle mode that emerges because of the long-range interactions in the gas. When adiabatically switching on the long-range interactions (*P*≠0), new quasi-particle modes of polaritonic character form, where intracavity photons are admixed to the recoil momentum states. A diagonalization of the Hamiltonian for *P*≠0 leads to the definition of a quasi-particle mode for the interacting system with annihilation and creation operators 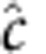 and 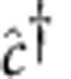, respectively (Supplementary Note 1). From our measurements (Fig. 2), we can directly extract the energy *ℏω*_s_ of this quasi-particle mode as a function of *P*/*P*_cr_. To this end, we fit a resonance curve of a damped harmonic oscillator to both sidebands of *S*(**k**_cb_,*ω*) (Supplementary Note 2), whose peak positions correspond to *ω*_s_, see Fig. 4. We observe the mode softening towards the critical point from both sides of the phase transition. The width *γ* of the sidebands is displayed in the lower panel of Fig. 4 and characterizes the damping of the quasi-particle mode. For our parameters, the main constituent of the quasi-particle mode is the atomic component, while the light field is only weakly admixed. We thus attribute the observed damping mainly to the decay of atomic momentum excitations. The finite decay rate *κ* of the cavity light field gives rise to an additional damping of the quasi-particle mode estimated to be only a few Hertz (Supplementary Note 1). The behaviour of the damping rate *γ* has been studied theoretically and originates from a resonant enhancement of the Beliaev damping of the checkerboard density wave^37^,^38^,^39^ and from finite temperature effects^36^. Our characterization of the quasi-particle mode is consistent with earlier measurements^8^,^29^, but now also extends into the organized phase because we can distinguish density fluctuations from a density modulation.

### Occupation of the quasi-particle mode

The occupation 
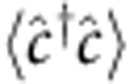
 of the quasi-particle mode can be extracted from the observed sideband asymmetry. As can be seen in Fig. 2, the red-shifted sideband dominates over the blue-shifted one. For a system in its ground state, only creation processes are possible, leading to a vanishing blue-shifted sideband. This has been used for thermometry of trapped ions and cavity optomechanical systems^40^,^41^. From the observation of the finite blue-shifted sideband in our experiment, we infer that the system is in a steady state close to its ground state. The continuous measurement process via cavity decay constantly creates and annihilates quasi-particles at rates 
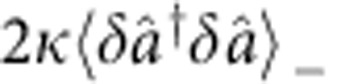
 and 
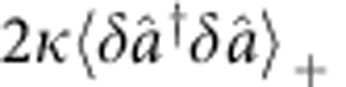
. Here 
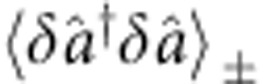
 is the integrated spectral weight of the blue (+) and red (−) sidebands, respectively. This measurement backaction effectively gives rise to a heating rate of the system, and thus to a finite occupation of the quasi-particle mode. At the same time, the finite decay rate *γ* of the quasi-particle mode changes this occupation. On one hand, quasi-particles will be annihilated because of this dissipation channel at rate 
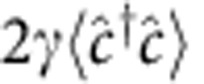
. On the other hand, it couples the quasi-particle mode to a thermal heat bath provided by the atomic cloud. This creates quasi-particles at rate 
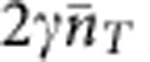
, where 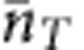 is the thermal occupation of the quasi-particle mode calculated from the Bose distribution function, and *T*=38(10) nK is the temperature of the BEC, measured independently from absorption images. In steady state, the different contributions are balanced according to the rate equation (see Supplementary Note 1 and Supplementary Fig. 1),


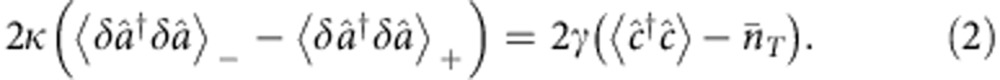


**Figure 5:**
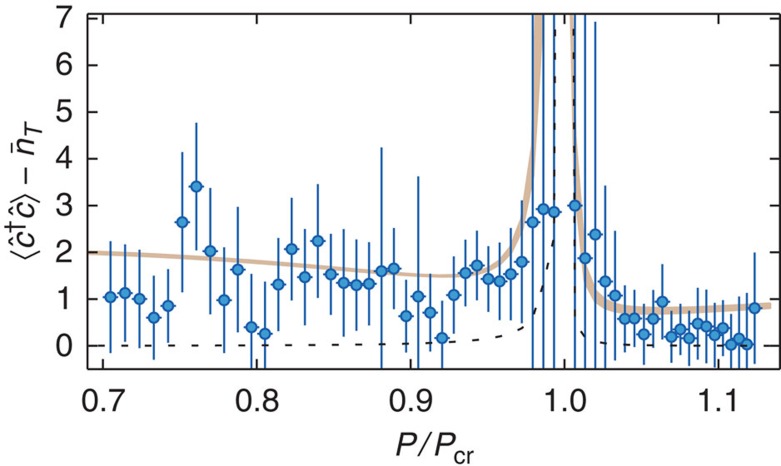
Number of quasi-particles. Number of quasi-particles 
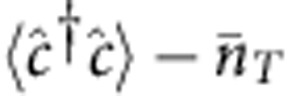
 as a function of relative pump power *P*/*P*_cr_, extracted from the sideband asymmetry in the dynamic structure factor. The grey-shaded area shows the result from an *ab initio* calculation of the expected quasi-particle mode occupation (Supplementary Notes 1 and 3), taking into account the experimental uncertainties in the determination of the coherent cavity field and the depth of the optical lattice resulting from the transverse pump field. Shown as black dashed line is the calculated thermal occupation 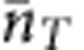 of the quasi-particle mode because of the finite temperature of the BEC for a temperature of 38 nK. Vertical and horizontal error bars indicate the statistical error (s.d.) reported from the fit, and the error in the determination of the critical point (s.d.), respectively. The strongly increased vertical error bars close to *P*/*P*_cr_=1 arise from the decreasing sideband asymmetry, while their individual errors stay roughly constant.

We can determine the occupation 
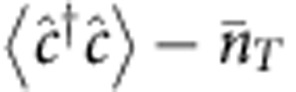
 of the quasi-particle mode, using the weights of the sidebands and the dissipation rates *γ* (empirical fit) and *κ* (see Fig. 5). We observe an average occupation of the quasi-particle mode on the level of only a few quanta. In the organized phase an increase in the mode occupation towards the critical point seems visible. The occupation is expected to diverge when approaching the critical point since the energy of the soft mode vanishes and the atomic damping rate goes to zero^38^,^39^. This situation is very similar to the enhanced thermal occupation of roton-like states predicted for dilute quantum gases with dipolar interactions at finite temperatures^9^.

## Discussion

We used vacuum-stimulated scattering of light to directly measure the dynamic structure factor of a quantum gas with cavity-mediated long-range interactions. Access to the dynamic structure factor allowed us to characterize the relevant quasi-particle mode while the system crossed a structural phase transition, to distinguish density modulation and density fluctuations, and to measure the critical exponents of the density fluctuations. We further extracted the finite occupancy of the quasi-particle mode under the influence of measurement backaction because of cavity decay and an atomic bath at finite temperature. While this measurement was motivated by the very specific set-up used to create cavity-mediated long-range interactions, an extension to more general settings seems possible. The approach of applying quantum optical methods based on strong matter–light interaction to the investigation of dilute ultracold gases offers unique possibilities for the nondestructive real-time investigation of quantum matter and its phase transitions^22^,^34^,^42^,^43^,^44^.

## Methods

### Experimental procedure

After centring an almost pure ^87^Rb BEC trapped in a crossed-beam dipole trap with respect to the TEM_00_ cavity mode, the transverse pump power *P*, at wavelength *λ*_p_=785.3 nm, is increased over 100 ms to a relative coupling strength of *P*/*P*_cr_≈0.46. Subsequently, the power *P* is linearly increased over 0.5 s to *P*/*P*_cr_≈1.38, while the stream of photons leaking out of the cavity is detected in a balanced heterodyne configuration using a local oscillator power of 2.2 mW and balanced photodiodes (Thorlabs PDB110A)^30^. The extracted quadratures at a beat frequency of 59.55 MHz are mixed down to 50 kHz, amplified, low-pass-filtered and digitized using high-speed analogue-to-digital converters with 2 μs resolution (National Instruments PCI-6132). The response of the heterodyne system is 2.2 V^2^ per cavity photon.

The temperature of the initially prepared BEC was determined from absorption images to be *T*=20(10) nK. The far-off resonant transverse pump beam heats the BEC during probing to a temperature of *T*=38(10) nK at the critical point. Residual atom loss of 26% during probing is included by rescaling the relative coupling axis according to the proportionality *P*_cr_∝*N*^−1^.

The phase transition point is characterized by a steep increase in the intracavity photon field. We fit the rise of the photon field once it has first exceeded a mean intracavity photon number of 4.5 with a saturation function *p*_0_ × (1−*t*_cr_/*t*)^*p*1^. With the extracted occurence time *t*_cr_ of the phase transition, we can convert the time axis into a relative coupling axis *P*/*P*_cr_. The relative statistical error of *P*_cr_ according to this procedure is given by 5 × 10^−4^, including intensity fluctuations of the transverse pump.

## Author contributions

R.L., R.M. and F.B. performed the experiments, R.L., R.M., F.B. and T.D. analysed the data. All authors contributed to the design of the experiments and the writing of the manuscript.

## Additional information

**How to cite this article:** Landig, R. *et al.* Measuring the dynamic structure factor of a quantum gas undergoing a structural phase transition. *Nat. Commun.* 6:7046 doi: 10.1038/ncomms8046 (2015).

## Supplementary Material

Supplementary Information Supplementary Figure 1, Supplementary Notes 1-3 and Supplementary References

## Figures and Tables

**Figure 1:**
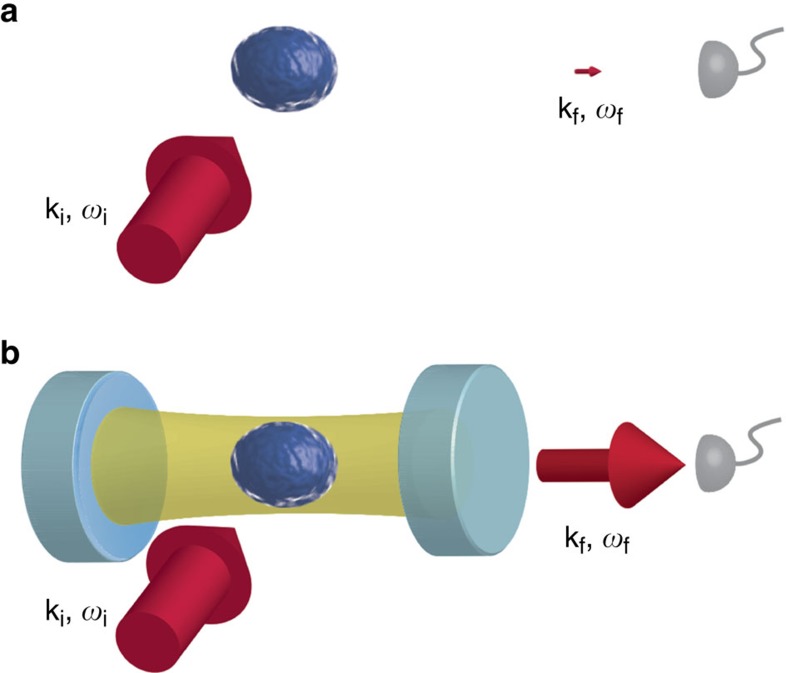
Scheme for measuring the dynamic structure factor in a quantum gas. (**a**) An incident laser beam (red) with wave vector **k**_i_ is spontaneously scattered at an atomic cloud (blue) into a free-space mode with wave vector **k**_f_. Analysing the scattered photons as a function of their frequency shift *ω*_f_−*ω*_i_ and magnitude yields the dynamic structure factor. The resulting signal for dilute quantum gases is vanishingly small, as indicated by the small size of the arrow pointing towards the detector. (**b**) Atoms placed into an optical high-finesse resonator (light blue) feel a strongly enhanced vacuum field (golden). Their spontaneous scattering rate into the mode **k**_f_ can hereby be increased by orders of magnitude, resulting in a detectable signal for the dynamic structure factor.
