# Supplementary material for: Measuring the dynamic structure factor of a quantum gas undergoing a structural phase transition
Source: Nat Commun. 2015 May 6;6:7046. doi: 10.1038/ncomms8046 (PMC4432596; doi:10.1038/ncomms8046)
Supplement: Supplementary Information — Supplementary Figure 1, Supplementary Notes 1-3 and Supplementary References [file ncomms8046-s1.pdf]

SUPPLEMENTARY FIGURES

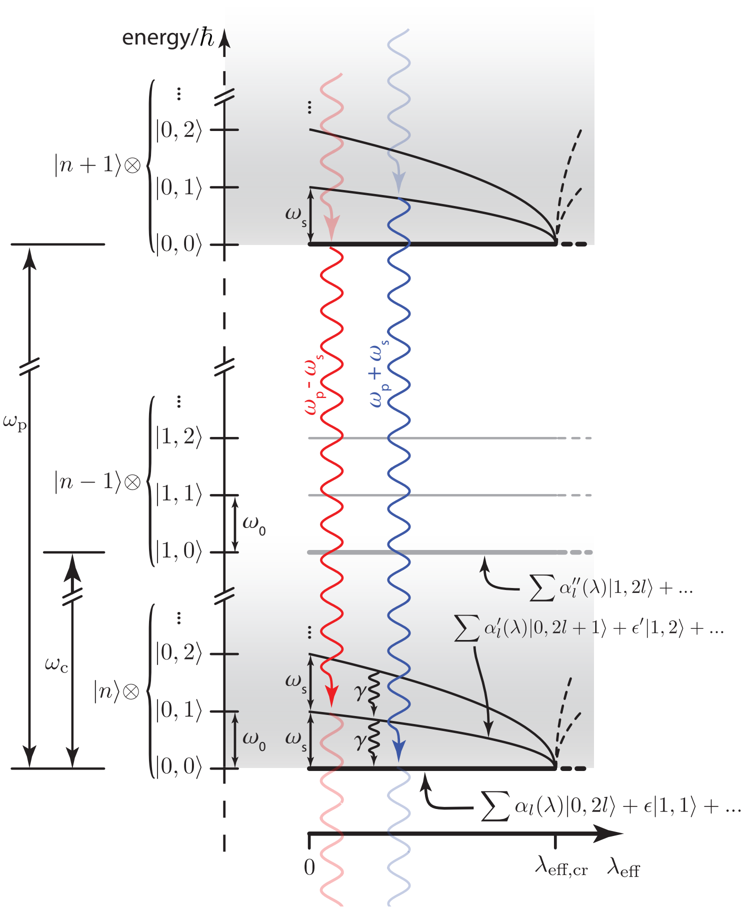

Supplementary Figure 1. **Energy diagram of the driven BEC-cavity system.** The bare states are denoted by $|n_p\rangle \otimes |n_c, n_a\rangle$, where n_p is the photon number of the coherent transverse pump field (shown are the manifolds of states for two different n_p), n_c is the intra-cavity photon number and n_a is the number of atoms with $(p_x, p_z) = (\pm\hbar k_x, \pm\hbar k_z)$. The coupling which conserves parity of $n_a + n_c$ softens the atomic excitation spectrum for increasing coupling strength λ_{eff}. Admixtures of states to the coupled states are indicated. At the critical coupling strength $\lambda_{\text{eff, cr}}$, the excitation gap $\hbar\omega_s$ vanishes, triggering the quantum phase transition to a self-organized atomic state. The finite cavity decay makes this phase transition of non-equilibrium nature as the corresponding decay processes (wiggling lines) give rise to a depletion of the ground state of the Hamiltonian system. The exiting cavity photons at frequency $\omega_p \pm \omega_s$ carry real-time information about the dynamics of the quantum many-body system, corresponding to the creation (red line) and annihilation (blue line) of quasi-particles. The collective momentum excitations can decay at rate γ, which results in an effective decrease of the annihilation processes via photon decay. This leads to the observed sideband asymmetry.

SUPPLEMENTARY NOTES

Supplementary Note 1: Theoretical description

Relation between the structure factor and the power spectral density in our system

We start with the definition of the dynamic structure factor as the spatial and temporal Fourier transform of the correlations of the density fluctuations $\delta\hat{\rho}(\mathbf{r}, t)$ [1]

$$S(\mathbf{k}, \omega) = \frac{1}{2\pi N/V} \int \mathrm{d}\mathbf{r}\mathrm{d}t\, e^{-i(\mathbf{kr}-\omega t)} \langle \delta\hat{\rho}(\mathbf{r}, t)\delta\hat{\rho}(0, 0)\rangle\,, \tag{1}$$

where V is the volume. With the relation $\rho(\mathbf{r}) = \frac{1}{V}\sum_k \rho_{\mathbf{k}} e^{i\mathbf{kr}}$ this can be rewritten in Fourier space as

$$S(\mathbf{k}, \omega) = \frac{1}{2\pi N} \int \mathrm{d}t\, e^{i\omega t} \langle \delta\hat{\rho}_{\mathbf{k}}(t)\delta\hat{\rho}_{-\mathbf{k}}(0)\rangle\,. \tag{2}$$

In steady state, the cavity light field operator $\hat{a}$ is given (neglecting small variations in the dispersive shift) by [2]

$$\hat{a} = \frac{\eta\hat{\Theta}}{\tilde{\Delta}_c - i\kappa}\,, \tag{3}$$

where the order parameter $\hat{\Theta} = \Theta_0 + \delta\hat{\Theta}$

$$\hat{\Theta} = \Theta_0 + \int \mathrm{d}^3\mathbf{r} \cos kx \cos kz \delta\hat{\rho}(\mathrm{r}, t) \tag{4}$$

$$= \Theta_0 + \frac{1}{4}\left(\delta\hat{\rho}_{(k,0,k)} + \delta\hat{\rho}_{(k,0,-k)} + \delta\hat{\rho}_{(-k,0,k)} + \delta\hat{\rho}_{(-k,0,-k)}\right)\,. \tag{5}$$

is written as a sum of a static density modulation $\Theta_0 = \langle\hat{\Theta}\rangle$ and time-dependent fluctuations. Here, the indices indicate the momentum along the x-, y-, respectively z-axis. We choose the coordinate system such that the cavity mode is oriented along the x-direction, and the transverse pump beam propagates along the z-direction.

We now expand the cavity light field around its mean field solution α, $\hat{a} = \alpha + \delta\hat{a}$ (and correspondingly the order parameter as $\hat{\Theta} = \Theta_0 + \delta\hat{\Theta}$), and use equation (3) to find for the

correlator

$$
\begin{aligned}
\langle \hat{a}^\dagger(t)\hat{a}(0)\rangle &= |\alpha|^2 + \langle \delta\hat{a}^\dagger(t)\delta\hat{a}(0)\rangle \\
&= \frac{\eta^2}{\kappa^2+\tilde{\Delta}_c^2}\left[\Theta_0^2 + \frac{1}{16}\langle \delta\hat{\rho}_{(k,0,k)}\delta\hat{\rho}_{(-k,0,-k)} + \delta\hat{\rho}_{(k,0,-k)}\delta\hat{\rho}_{(-k,0,k)}\right. \\
&\left. +\delta\hat{\rho}_{(-k,0,k)}\delta\hat{\rho}_{(k,0,-k)} + \delta\hat{\rho}_{(-k,0,-k)}\delta\hat{\rho}_{(k,0,k)}\rangle\right] \\
&= \frac{\eta^2}{\kappa^2+\tilde{\Delta}_c^2}\left[\Theta_0^2 + \frac{1}{4}\langle \delta\hat{\rho}_{(k,0,k)}\delta\hat{\rho}_{(-k,0,-k)}\rangle\right] .
\end{aligned}
\tag{6}
$$

Here, we made use of the fact that $\langle \delta\hat{\rho}_{\mathbf{q}}(t)\delta\hat{\rho}_{\mathbf{q}'}(0)\rangle = 0$, unless $\mathbf{q} = -\mathbf{q}'$, and that the system is symmetric under the transformations $x \to -x$ and $z \to -z$ due to the involved standing waves. Since the Fourier transform of the temporal correlation function $\langle \hat{a}^\dagger(t)\hat{a}(0)\rangle$ is the power spectral density of the intra-cavity light field, we finally obtain a direct relation between the dynamic structure factor and the spectrum of the intra-cavity light field:

$$
PSD(\omega) = \int \mathrm{d}t\, e^{-i\omega t}\langle \hat{a}^\dagger(t)\hat{a}(0)\rangle \tag{7}
$$

$$
= \frac{\eta^2}{\kappa^2+\tilde{\Delta}_c^2}\left[2\pi\Theta_0^2\delta(\omega) + \frac{2\pi N}{4}S(\mathbf{k}_{\mathrm{cb}},\omega)\right] . \tag{8}
$$

The power spectral density $PSD(\omega)$ thus quantifies both the checkerboard density modulation (first term, zero frequency bin) and the dynamic structure factor at the wavevector $\mathbf{k}_{\mathrm{cb}} = (\pm k, 0, \pm k)$ (second term).

Finally we can extract the dynamic structure factor as

$$
S(\mathbf{k}_{\mathrm{cb}},\omega) = \frac{\kappa^2+\tilde{\Delta}_c^2}{\eta^2}\frac{4}{N}\left(\frac{1}{2\pi}PSD(\omega) - |\alpha|^2\delta(\omega)\right) . \tag{9}
$$

For the experimentally relevant case of two nearly degenerate, circularly polarized cavity modes, we find accordingly

$$
S(\mathbf{k}_{\mathrm{cb}},\omega) = \frac{\kappa^2+\tilde{\Delta}_c^{(\mathrm{eff})2}}{\eta_1^2+\eta_2^2}\frac{4}{N}\left(\frac{1}{2\pi}PSD(\omega) - |\alpha|^2\delta(\omega)\right) , \tag{10}
$$

where the definitions of η_i and $\tilde{\Delta}_{c,\mathrm{eff}}$ are given below, and α and $PSD(\omega)$ refer to the according superposition of the bare cavity modes coupling to the atoms.

Derivation of the Hamiltonian describing the fluctuations of the system

In this section we derive an effective Hamiltonian describing the fluctuations of the system starting from the many-body Hamiltonian,

$$\hat{H}_{\rm mb} = \hat{H}_{\rm c} + \hat{H}_{\rm a} + \hat{H}_{\text{a-c}} + \hat{H}_{\rm SB}\,, \tag{11}$$

with

$$\begin{aligned}
\hat{H}_{\rm c} &= -\hbar\Delta_{\rm c}\sum_{i=1}^{2}\hat{a}_i^\dagger\hat{a}_i \\
\hat{H}_{\rm a} &= \int {\rm d}^3r\hat{\Psi}^\dagger(\mathbf{r})\left[\frac{\mathbf{p}^2}{2m} + V_{\rm p}\cos^2(kz) + \frac{g}{2}\hat{\Psi}^\dagger(\mathbf{r})\hat{\Psi}(\mathbf{r})\right]\hat{\Psi}(\mathbf{r}) \\
\hat{H}_{\text{a-c}} &= \sum_{i=1}^{2}\int {\rm d}^3r\hat{\Psi}^\dagger(\mathbf{r})\left[\hbar\eta_i\cos(kx)\cos(kz)(\hat{a}_i+\hat{a}_i^\dagger) + \hbar U_0^i\cos^2(kx)\hat{a}_i^\dagger\hat{a}_i\right]\hat{\Psi}(\mathbf{r}) \\
\hat{H}_{\rm SB} &= \hbar\zeta\sum_{i=1}^{2}\eta_i(\hat{a}_i+\hat{a}_i^\dagger)\,.
\end{aligned} \tag{12}$$

Here we take into account that the cavity is supporting two degenerate (neglecting a small birefringence on the order of the cavity line width), circularly polarized cavity modes with annihilation operators $(\hat{a}_1, \hat{a}_2)$, which are coupled to the atoms with two-photon Rabi frequencies (η_1, η_2) [3]. The maximum dispersive shift of the two cavity modes due to the dispersive coupling of a single atom is described by (U_0^1, U_0^2). A symmetry breaking term $\hat{H}_{\rm SB}$ is introduced, which is proportional to a real-valued effective cavity drive amplitude ζ (see also definitions in [2]).

Following references [3, 4], we expand the atomic and cavity field operators $(\hat{\Psi}, \hat{a}_i)$ around their mean-field values (ψ_0, α_0^i),

$$\hat{\Psi} = (\sqrt{N}\psi_0 + \delta\hat{\Psi})e^{-it\mu_0/\hbar} \tag{13}$$

$$\hat{a}_i = \alpha_0^i + \delta\hat{a}_i\,. \tag{14}$$

Expanding Hamiltonian $\hat{H}_{\rm mb}$, Eq. (11), in the limit $|\Delta_c| \gg \kappa$ up to second order in the

fluctuation operators $(\delta\hat{\Psi}, \delta\hat{a}_i)$, we find the quadratic Hamiltonian

$$\hat{H}^{(2)} = \hat{H}_0 + \hbar \sum_{i=1}^{2} \left(\eta_i \delta\hat{\Theta} + U_0^i \alpha_0^i \delta\hat{B}\right)(\delta\hat{a}_i + \delta\hat{a}_i^\dagger) + \hbar\tilde{\Delta}_c^i \delta\hat{a}_i^\dagger \delta\hat{a}_i \,, \tag{15}$$

with

$$\begin{aligned} \hat{H}_0 = \int d^3r \; \delta\hat{\Psi}^\dagger \Bigg[\frac{-\hbar^2}{2m}(\partial_x^2 + \partial_z^2) + V_{\rm p}(z) + \hbar \sum_{i=1}^{2} \eta_i(x,z)(\alpha_0^i + \alpha_0^{i*}) \\ + \hbar \sum_{i=1}^{2} U_0^i(x)|\alpha_0^i|^2 \Bigg] \delta\hat{\Psi} + \frac{1}{2}\, g_{\rm 2D}\, \psi_0^2 \left(\delta\hat{\Psi}^2 + (\delta\hat{\Psi}^\dagger)^2\right) + 2g_{\rm 2D}|\psi_0|^2 \delta\hat{\Psi}^\dagger \delta\hat{\Psi} \,, \end{aligned} \tag{16}$$

where $\tilde{\Delta}_c^i = \Delta_c - \mathcal{B}_0 U_0^i$. We introduced here the spatially dependent classical lattice potential $V_p(z) = V_p \cos^2(kz)$ of the transverse pump, the spatially dependent two-photon Rabi frequencies $\eta_i(x,z) = \frac{\Omega_p g_0^{(i)}}{\Delta_a} \cos(kx)\cos(kz)$ with maximum pump Rabi frequency Ω_p, detuning $\Delta_a = \omega_p - \omega_a$ between atomic resonance frequency and pump light frequency, and single-atom coupling strengths $g_0^{(i)}$. Further, the light shift per photon is $U_0^i(x) = \frac{g_0^{(i)2}}{\Delta_a} \cos^2(kx)$. Along the third direction, y, we assume a homogeneous system and use the according contact interaction strength g_{2D} [5]. We introduced the definition $\delta\hat{\Theta} = \sqrt{N} \int \mathrm{d}^3r(\delta\hat{\Psi}^\dagger + \delta\hat{\Psi}) \cos(kx)\cos(kz)\psi_0$ of the fluctuations of the order parameter $\hat{\Theta} = \int \mathrm{d}^3r \hat{\Psi}^\dagger \hat{\Psi} \cos(kx)\cos(kz)$ around the mean field value $\Theta_0 = \int \mathrm{d}^3r |\psi_0|^2 \cos(kx)\cos(kz)$. In a similar way, we define the fluctuation operator $\delta\hat{\mathcal{B}} = \sqrt{N} \int \mathrm{d}^3r(\delta\hat{\Psi}^\dagger + \delta\hat{\Psi}) \cos(kx)^2 \psi_0$ of the bunching operator $\hat{\mathcal{B}} = \int \mathrm{d}^3r \hat{\Psi}^\dagger \hat{\Psi} \cos(kx)^2$ around its mean-field value $\mathcal{B}_0 = \int \mathrm{d}^3r |\psi_0|^2 \cos(kx)^2$.

The mean-field values are determined by the corresponding Gross-Pitaevskii equation derived from $\hat{H}^{(2)}$:

$$\begin{aligned} \mu_0 \psi_0 = \Bigg(-\frac{\hbar^2}{2m}(\partial_x^2 + \partial_z^2) + V_p(z) + \hbar \sum_{i=1}^{2} U_0^i(x)|\alpha_0^i|^2 \\ + \hbar \sum_{i=1}^{2} \eta_i(x,z)(\alpha_0^i + \alpha_0^{i*} + g_{2D}|\psi_0|^2) \Bigg) \psi_0(x,z) \,, \end{aligned} \tag{17}$$

with $\alpha_0^i = \frac{\eta_i \Theta_0}{\tilde{\Delta}_c^i + i\kappa}$, assuming adiabatic following of the cavity field.

To find the collective excitations of the system around the mean-field solution, we expand $\delta\hat{\Psi}$ in Bogoliubov modes $\hat{h}_j$ (which diagonalize H_0 with eigenenergies E_j) with amplitudes

$u_j(\mathbf{r})$ and $v_j(\mathbf{r})$,

$$\delta\hat{\Psi}(\mathbf{r}) = \sum_j \left(u_j(\mathbf{r})\hat{h}_j + v_j^*(\mathbf{r})\hat{h}_j^\dagger \right) , \tag{18}$$

resulting in

$$\begin{aligned}\hat{H}^{(2)} =& \sum_j \left[E_j \hat{h}_j^\dagger \hat{h}_j + \hbar \sum_{i=1}^{2} \eta_i \left(\hat{h}_j \sqrt{N\chi_j^{i*}} + \text{h.c.} \right) \left(\delta\hat{a}_i^\dagger + \delta\hat{a}_i \right) \right] \\ &+ \hbar \sum_{i=1}^{2} -\tilde{\Delta}_c^i \delta\hat{a}_i^\dagger \delta\hat{a}_i \, .\end{aligned} \tag{19}$$

Here, we introduced interaction matrix elements $\chi_j^i = \langle \psi_0 | \cos(kx)\cos(kz) + \frac{\Theta_0 U_0^i}{\tilde{\Delta}_c^i} \cos^2(kx) | u_j + v_j \rangle$, describing the overlap between the ground state wave function and the Bogoliubov excitations as presented in reference [3]. From numerical calculations we know that only a single Bogoliubov mode $\hat{h}_0 = \hat{h}$ with energy $E_0 = E$ is dominantly contributing via the matrix elements $\chi_0^i = \chi^i$, and all other matrix elements are suppressed by more than two orders of magnitude [3]. This leads to

$$\hat{H}^{(2)} = E\,\hat{h}^\dagger\hat{h} + \hbar\sum_{i=1}^{2} -\tilde{\Delta}_c^i\,\delta\hat{a}_i^\dagger\delta\hat{a}_i + \hbar\sum_{i=1}^{2}\eta_i\sqrt{N}\left(\hat{h}\sqrt{\chi^{i*}} + \hat{h}^\dagger\sqrt{\chi^i}\right)\left(\delta\hat{a}_i^\dagger + \delta\hat{a}_i\right) . \tag{20}$$

If we now use the definitions $\omega_0 = E/\hbar$ for the bare energy of the uncoupled system, $\lambda_i = \sqrt{N}\eta_i\sqrt{|\chi^i|}$ for the coupling strength and $\delta\hat{b} = \hat{h}e^{i\phi_i}$ for the collective atomic fluctuations (using $\sqrt{\chi^i} = \sqrt{|\chi^i|}e^{i\phi_i} \approx \sqrt{|\chi^i|}$, where $\phi_i \approx 0$, i.e. $\phi_1 \approx \phi_2$), we finally arrive at the fluctuation Hamiltonian

$$\hat{H} = \hbar\omega_0\delta\hat{b}^\dagger\delta\hat{b} + \hbar\sum_{i=1}^{2} -\tilde{\Delta}_c^i\delta\hat{a}_i^\dagger\delta\hat{a}_i + \hbar\sum_{i=1}^{2}\lambda_i\left(\delta\hat{a}_i + \delta\hat{a}_i^\dagger\right)\left(\delta\hat{b} + \delta\hat{b}^\dagger\right) . \tag{21}$$

In order to further simplify this expression, we apply a transformation which reduces the Hamiltonian describing two cavity modes to a Hamiltonian involving coupling to only one effective cavity mode. We introduce two new cavity modes with fluctuation operators $(\hat{o}_1, \hat{o}_2)$, which are a linear superposition of the original modes $(\delta\hat{a}_1, \delta\hat{a}_2)$

$$\delta\hat{a}_1 = A\hat{o}_1 + B\hat{o}_2 \tag{22}$$

$$\delta\hat{a}_2 = C\hat{o}_1 + D\hat{o}_2 \, . \tag{23}$$

Applying this transformation and requesting that the new operators obey bosonic commutation relations ($[\hat{o}_1, \hat{o}_1^\dagger] = 1, [\hat{o}_2, \hat{o}_2^\dagger] = 1$), that mixed terms of the new operators vanish, and that mode $\hat{o}_2$ decouples from the atoms, we find the coefficients

$$A = \frac{\lambda_1 \tilde{\Delta}_c^{(2)}}{\lambda_2 \tilde{\Delta}_c^{(1)}} C \,, \quad B = \frac{\sqrt{\left(\frac{\lambda_1 \tilde{\Delta}_c^{(2)}}{\lambda_2 \tilde{\Delta}_c^{(1)}}\right)^2 + 1}}{1 + \frac{\lambda_1^2 \tilde{\Delta}_c^{(2)}}{\lambda_2^2 \tilde{\Delta}_c^{(1)}}} \,, \quad C = -\frac{\lambda_1}{\lambda_2} B \,, \quad D = \frac{\sqrt{\left(\frac{\lambda_1}{\lambda_2}\right)^2 + 1}}{1 + \frac{\lambda_1^2 \tilde{\Delta}_c^{(2)}}{\lambda_2^2 \tilde{\Delta}_c^{(1)}}} \,. \tag{24}$$

Inserting these coefficients into $\hat{H}$ yields

$$\begin{aligned} \hat{H} = &- \hbar \frac{(\lambda_1^2 + \lambda_2^2) \tilde{\Delta}_c^{(1)} \tilde{\Delta}_c^{(2)}}{\lambda_1^2 \tilde{\Delta}_c^{(2)} + \lambda_2^2 \tilde{\Delta}_c^{(1)}} \hat{o}_1^\dagger \hat{o}_1 - \hbar \frac{\lambda_1^2 \tilde{\Delta}_c^{(2)2} + \lambda_2^2 \tilde{\Delta}_c^{(1)2}}{\lambda_1^2 \tilde{\Delta}_c^{(2)} + \lambda_2^2 \tilde{\Delta}_c^{(1)}} \hat{o}_2^\dagger \hat{o}_2 \\ &+ \hbar \sqrt{\lambda_1^2 + \lambda_2^2} (\hat{o}_1 + \hat{o}_1^\dagger)(\delta \hat{b} + \delta \hat{b}^\dagger) + \hbar \omega_0 \delta \hat{b}^\dagger \delta \hat{b} \,. \end{aligned} \tag{25}$$

The mode $\hat{o}_2$ is now decoupled from atomic motion and we can write the effective fluctuation Hamiltonian for the system

$$\hat{H}/\hbar = \omega_0 \delta \hat{b}^\dagger \delta \hat{b} - \tilde{\Delta}_{c,\text{eff}} \delta \hat{a}^\dagger \delta \hat{a} + \lambda_{\text{eff}} (\delta \hat{a}^\dagger + \delta \hat{a})(\delta \hat{b}^\dagger + \delta \hat{b}) \,, \tag{26}$$

where we used

$$\delta \hat{a} = \hat{o}_1 \tag{27}$$

$$\tilde{\Delta}_{c,\text{eff}} = \frac{(\lambda_1^2 + \lambda_2^2) \tilde{\Delta}_c^{(1)} \tilde{\Delta}_c^{(2)}}{\lambda_1^2 \tilde{\Delta}_c^{(2)} + \lambda_2^2 \tilde{\Delta}_c^{(1)}} \tag{28}$$

$$\lambda_{\text{eff}} = \sqrt{\lambda_1^2 + \lambda_2^2} \,. \tag{29}$$

Langevin description of the coupled system

The goal of this section is to derive coupled quantum Langevin equations effectively describing our system. The hierarchy of the relevant energy scales determines their derivation. The fastest timescale in the system is given by the cavity field decay at rate $\kappa \approx 2\pi \times 1.25$ MHz, followed by the variable coupling rate λ_{eff}, which reaches $\approx 2\pi \times 100$ kHz at the critical point, and finally the damping rate γ of the atomic excitation, which is on the order of a few hundred Hertz [2]. Following this hierarchy, we first couple the damped optical cavity mode to the Bogoliubov mode $\delta \hat{b}$. This results in new polariton modes of the system

which describe the quasi-particles of the long-range interacting system. Only thereafter we introduce the damping of the atomic polariton mode at rate γ. This approach is justified as the admixture of the cavity mode to the polariton is very small ($\omega_0/\tilde{\Delta}_{c,\text{eff}} \ll 1$).

We start with Hamiltonian Eq. (26),

$$\hat{H}/\hbar = \omega_0 \delta\hat{b}^\dagger \delta\hat{b} + \tilde{\Delta}_{c,\text{eff}} \delta\hat{a}^\dagger \delta\hat{a} + \lambda_{\text{eff}}(\delta\hat{a}^\dagger + \delta\hat{a})(\delta\hat{b}^\dagger + \delta\hat{b}) \tag{30}$$

The corresponding quantum Langevin equation with cavity field damping rate κ read

$$\delta\dot{\hat{a}} = (-i\tilde{\Delta}_{c,\text{eff}} - \kappa)\delta\hat{a} - i\lambda_{\text{eff}}(\delta\hat{b} + \delta\hat{b}) + \sqrt{2\kappa}\hat{a}_{\text{in}} \tag{31}$$

$$\delta\dot{\hat{b}} = -i\omega_0\delta\hat{b} - i\lambda_{\text{eff}}(\delta\hat{a} + \delta\hat{a}^\dagger) \tag{32}$$

The bosonic operator $\hat{a}_{\text{in}}$ in Eq. (31), describes vacuum input fluctuations of the surrounding electromagnetic field modes which are characterized by the correlation functions $\langle \hat{a}_{\text{in}}(t)\hat{a}^\dagger_{\text{in}}(t')\rangle = \delta(t-t')$ and $\langle \hat{a}^\dagger_{\text{in}}(t)\hat{a}_{\text{in}}(t')\rangle = 0$.

In order to find the new eigenmodes (polaritons) of the coupled system we rewrite the Langevin equations in matrix form,

$$\frac{d}{dt}\begin{pmatrix} \delta\hat{a} \\ \delta\hat{a}^\dagger \\ \delta\hat{b} \\ \delta\hat{b}^\dagger \end{pmatrix} = M_0 \begin{pmatrix} \delta\hat{a} \\ \delta\hat{a}^\dagger \\ \delta\hat{b} \\ \delta\hat{b}^\dagger \end{pmatrix} + \sqrt{2\kappa}\begin{pmatrix} \hat{a}_{\text{in}} \\ \hat{a}^\dagger_{\text{in}} \\ 0 \\ 0 \end{pmatrix} \tag{33}$$

with the matrix

$$M_0 = \begin{pmatrix} -i\tilde{\Delta}_{c,\text{eff}} - \kappa & 0 & -i\lambda_{\text{eff}} & -i\lambda_{\text{eff}} \\ 0 & i\tilde{\Delta}_{c,\text{eff}} - \kappa & i\lambda_{\text{eff}} & i\lambda_{\text{eff}} \\ -i\lambda_{\text{eff}} & -i\lambda_{\text{eff}} & -i\omega_0 & 0 \\ i\lambda_{\text{eff}} & i\lambda_{\text{eff}} & 0 & -i\omega_0 \end{pmatrix} \tag{34}$$

Diagonalization of M_0 via the transformation

$$S^{-1}M_0S = D = \begin{pmatrix} -i\tilde{\Delta}_{c,\text{eff}} - \kappa & 0 & 0 & 0 \\ 0 & i\tilde{\Delta}_{c,\text{eff}} - \kappa & 0 & 0 \\ 0 & 0 & -i\omega_s & 0 \\ 0 & 0 & 0 & i\omega_s \end{pmatrix} \tag{35}$$

with, neglecting terms of order $\frac{\omega_0}{\omega}$,

$$S = \begin{pmatrix} 1 & 0 & \frac{-\lambda_{\text{eff}}}{\tilde{\Delta}_{c,\text{eff}}-i\kappa}\sqrt{\frac{\omega_0}{\omega_s}} & \frac{-\lambda_{\text{eff}}}{\tilde{\Delta}_{c,\text{eff}}-i\kappa}\sqrt{\frac{\omega_0}{\omega_s}} \\ 0 & 1 & \frac{-\lambda_{\text{eff}}}{\tilde{\Delta}_{c,\text{eff}}+i\kappa}\sqrt{\frac{\omega_0}{\omega_s}} & \frac{-\lambda_{\text{eff}}}{\tilde{\Delta}_{c,\text{eff}}+i\kappa}\sqrt{\frac{\omega_0}{\omega_s}} \\ \frac{-i\lambda_{\text{eff}}}{i\tilde{\Delta}_{c,\text{eff}}+\kappa} & \frac{-i\lambda_{\text{eff}}}{-i\tilde{\Delta}_{c,\text{eff}}+\kappa} & \frac{1+\omega_s/\omega_0}{2}\sqrt{\frac{\omega_0}{\omega_s}} & \frac{1-\omega_s/\omega_0}{2}\sqrt{\frac{\omega_0}{\omega_s}} \\ \frac{i\lambda_{\text{eff}}}{i\tilde{\Delta}_{c,\text{eff}}+\kappa} & \frac{i\lambda_{\text{eff}}}{-i\tilde{\Delta}_{c,\text{eff}}+\kappa} & \frac{1-\omega_s/\omega_0}{2}\sqrt{\frac{\omega_0}{\omega_s}} & \frac{1+\omega_s/\omega_0}{2}\sqrt{\frac{\omega_0}{\omega_s}} \end{pmatrix} \tag{36}$$

and

$$S^{-1} = \begin{pmatrix} 1 & 0 & \frac{i\lambda_{\text{eff}}}{i\tilde{\Delta}_{c,\text{eff}}+\kappa} & \frac{i\lambda_{\text{eff}}}{i\tilde{\Delta}_{c,\text{eff}}+\kappa} \\ 0 & 1 & \frac{-i\lambda_{\text{eff}}}{-i\tilde{\Delta}_{c,\text{eff}}+\kappa} & \frac{-i\lambda_{\text{eff}}}{-i\tilde{\Delta}_{c,\text{eff}}+\kappa} \\ \frac{-\lambda_{\text{eff}}}{\tilde{\Delta}_{c,\text{eff}}-i\kappa}\sqrt{\frac{\omega_0}{\omega_s}} & \frac{\lambda_{\text{eff}}}{\tilde{\Delta}_{c,\text{eff}}+i\kappa}\sqrt{\frac{\omega_0}{\omega_s}} & \frac{1+\omega_0/\omega_s}{2}\sqrt{\frac{\omega_s}{\omega_0}} & \frac{1-\omega_0/\omega_s}{2}\sqrt{\frac{\omega_s}{\omega_0}} \\ \frac{\lambda_{\text{eff}}}{\tilde{\Delta}_{c,\text{eff}}-i\kappa}\sqrt{\frac{\omega_0}{\omega_s}} & \frac{-\lambda_{\text{eff}}}{\tilde{\Delta}_{c,\text{eff}}+i\kappa}\sqrt{\frac{\omega_0}{\omega_s}} & \frac{1-\omega_0/\omega_s}{2}\sqrt{\frac{\omega_s}{\omega_0}} & \frac{1+\omega_0/\omega_s}{2}\sqrt{\frac{\omega_s}{\omega_0}} \end{pmatrix} \tag{37}$$

allows us to define the polariton mode operators $\hat{d}$ and $\hat{c}$. Here,

$$\omega_s = \omega_0\sqrt{1 - (\lambda_{\text{eff}}/\lambda_{\text{eff, cr}})^2} \tag{38}$$

is the eigenfrequency of the quasi-particle mode we are interested in [6]. Note that S^{-1} is normalized such that the bosonic commutation relations for $\hat{d}^\dagger$ and $\hat{c}^\dagger$ defined as

$$\begin{pmatrix} \hat{d} \\ \hat{d}^\dagger \\ \hat{c} \\ \hat{c}^\dagger \end{pmatrix} = S^{-1}\begin{pmatrix} \delta\hat{a} \\ \delta\hat{a}^\dagger \\ \delta\hat{b} \\ \delta\hat{b}^\dagger \end{pmatrix} \tag{39}$$

are valid. This way we achieve the following expressions for the operators $\hat{d}$ ($\hat{c}$), an-

nihilating an excitation in the optical (atomic) polariton mode with eigenfrequency $\tilde{\Delta}_{c,\text{eff}}$ (ω_s):

$$
\begin{aligned}
\hat{d} &= \delta\hat{a} + \frac{i\lambda_{\text{eff}}}{i\tilde{\Delta}_{c,\text{eff}} + \kappa}(\delta\hat{b} + \delta\hat{b}^\dagger) \\
\hat{c} &= \sqrt{\frac{\omega_0}{\omega_s}}\lambda_{\text{eff}} \left(\frac{-\delta\hat{a}}{\tilde{\Delta}_{c,\text{eff}} - i\kappa} + \frac{\delta\hat{a}^\dagger}{\tilde{\Delta}_{c,\text{eff}} + i\kappa} \right) \\
&\quad + \frac{1}{2}\sqrt{\frac{\omega_s}{\omega_0}} \left((1 + \sqrt{\frac{\omega_0}{\omega_s}})\delta\hat{b} + (1 - \sqrt{\frac{\omega_0}{\omega_s}})\delta\hat{b}^\dagger \right).
\end{aligned}
\tag{40}
$$

Again, this is correct up to terms of order $\omega_0/\tilde{\Delta}_{c,\text{eff}}$. For $\lambda_{\text{eff}} \to 0$ these equations yield $\hat{d} \to \delta\hat{a}$ and $\hat{c} \to \delta\hat{b}$, such that the polariton modes evolve into the bare modes for vanishing coupling.

Langevin equations for the polariton modes

We now can write down the Langevin equations for the polariton modes. Starting from equation (33) we use

$$
\frac{d}{dt}\begin{pmatrix} \hat{d} \\ \hat{d}^\dagger \\ \hat{c} \\ \hat{c}^\dagger \end{pmatrix} = \frac{d}{dt}S^{-1}\begin{pmatrix} \delta\hat{a} \\ \delta\hat{a}^\dagger \\ \delta\hat{b} \\ \delta\hat{b}^\dagger \end{pmatrix} = S^{-1}M_0SS^{-1}\begin{pmatrix} \delta\hat{a} \\ \delta\hat{a}^\dagger \\ \delta\hat{b} \\ \delta\hat{b}^\dagger \end{pmatrix} + \sqrt{2\kappa}S^{-1}\begin{pmatrix} \hat{a}_{\text{in}} \\ \hat{a}_{\text{in}}^\dagger \\ 0 \\ 0 \end{pmatrix}
\tag{41}
$$

to find

$$
\dot{\hat{d}} = (-i\tilde{\Delta}_{c,\text{eff}} - \kappa)\hat{d} + \sqrt{2\kappa}\hat{a}_{\text{in}} \tag{42}
$$

$$
\dot{\hat{c}} = -i\omega_s\hat{c} + \sqrt{2\kappa}\sqrt{\frac{\omega_0}{\omega_s}}\lambda_{\text{eff}}\left(\frac{-\hat{a}_{\text{in}}}{\tilde{\Delta}_{c,\text{eff}} - i\kappa} + \frac{\hat{a}_{\text{in}}^\dagger}{\tilde{\Delta}_{c,\text{eff}} + i\kappa} \right) \tag{43}
$$

The input noise terms in the equation for $\hat{c}$ correspond to the quantum backaction: although the cavity bath is at $T = 0$, the c-mode is driven by quantum noise from the open channel.

Damping of the c-mode

We now formally introduce a damping of the atomic polariton mode which effectively models its collisional interaction with the surrounding Bogoliubov modes of the atomic cloud. In principle, this mode is also damped by the cavity decay channel. However, since $\omega_0/\tilde{\Delta}_{c,\text{eff}} \ll 1$, this damping rate is on the order of a few Hertz and will be neglected here. We define input noise operators with correlation functions $\langle \hat{c}_{\text{in}}(t)\hat{c}^{\dagger}_{\text{in}}(t')\rangle = \delta(t-t')(1+\bar{n}_T)$ and $\langle \hat{c}^{\dagger}_{\text{in}}(t)\hat{c}_{\text{in}}(t')\rangle = \delta(t-t')\bar{n}_T$. The thermal occupation number $\bar{n}_T$ of the atomic polariton mode is modeled by the Bose distribution function, evaluated at the soft mode frequency ω_s of the coupled system. Accordingly, the Langevin equation (43) becomes

$$\dot{\hat{c}} = (-i\omega_s - \gamma)\hat{c} + \sqrt{2\gamma}\hat{c}_{\text{in}} + \sqrt{2\kappa}\sqrt{\frac{\omega_0}{\omega_s}}\lambda_{\text{eff}}\left(\frac{-\hat{a}_{\text{in}}}{\tilde{\Delta}_{c,\text{eff}} - i\kappa} + \frac{\hat{a}^{\dagger}_{\text{in}}}{\tilde{\Delta}_{c,\text{eff}} + i\kappa}\right) \tag{44}$$

Occupation number of the c-mode

With this result, we can now calculate the expectation value $\langle \hat{c}^{\dagger}(t)\hat{c}(t)\rangle$ for the number of quasi-particles. From the solution of Eq. (44),

$$\hat{c}(t) = \int_0^t e^{(-i\omega_s-\gamma)(t-t')}\left(\sqrt{2\gamma}\hat{c}_{\text{in}} + \sqrt{2\kappa}\sqrt{\frac{\omega_0}{\omega_s}}\lambda_{\text{eff}}\left(\frac{-\hat{a}_{\text{in}}}{\tilde{\Delta}_{c,\text{eff}} - i\kappa} + \frac{\hat{a}^{\dagger}_{\text{in}}}{\tilde{\Delta}_{c,\text{eff}} + i\kappa}\right)\right), \tag{45}$$

we find

$$\langle \hat{c}^{\dagger}(t)\hat{c}(t)\rangle = \bar{n}_T + \frac{2\kappa}{2\gamma}\frac{\omega_0}{\omega_s}\frac{\lambda^2_{\text{eff}}}{\tilde{\Delta}^2_{c,\text{eff}} + \kappa^2}, \tag{46}$$

which diverges towards the critical point with an exponent 0.5 as a function of $1 - \lambda_{\text{eff}}/\lambda_{\text{cr}}$.

Spectrum of the light field

The main observable in our experiment is the photon spectrum of the light field leaking out of the cavity. In order to find a relation between this spectrum and the $\hat{c}$- and $\hat{d}$-mode, we apply a backtransformation using the matrix S, and move to frequency space (for the definition of the Fourier transformation see [2]):

$$\delta\hat{a}(\omega) = \hat{d}(\omega) - \lambda_{\text{eff}}\frac{\tilde{\Delta}_{c,\text{eff}} + i\kappa}{\tilde{\Delta}^2_{c,\text{eff}} + \kappa^2}\sqrt{\frac{\omega_0}{\omega_s}}\left(\hat{c}(\omega) + \hat{c}^\dagger(-\omega)\right) \tag{47}$$

$$\delta\hat{a}^\dagger(\omega) = \hat{d}^\dagger(\omega) - \lambda_{\text{eff}}\frac{\tilde{\Delta}_{c,\text{eff}} + i\kappa}{\tilde{\Delta}^2_{c,\text{eff}} + \kappa^2}\sqrt{\frac{\omega_0}{\omega_s}}\left(\hat{c}^\dagger(\omega) + \hat{c}(-\omega)\right) \tag{48}$$

Using the Langevin equation in Fourier space [7], the expectation value of the cavity output photon spectrum can be written as

$$\begin{aligned}
\langle\delta\hat{a}^\dagger(\omega)\delta\hat{a}(\omega')\rangle &= \langle\hat{d}^\dagger(\omega)\hat{d}(\omega')\rangle + \frac{\lambda^2_{\text{eff}}}{\tilde{\Delta}^2_{c,\text{eff}} + \kappa^2}\frac{\omega_0}{\omega_s}\left(\langle\hat{c}^\dagger(\omega)\hat{c}(\omega')\rangle + \langle\hat{c}(-\omega)\hat{c}^\dagger(-\omega')\rangle\right) \\
&= \frac{\lambda^2_{\text{eff}}}{\tilde{\Delta}^2_{c,\text{eff}} + \kappa^2}\frac{\omega_0}{\omega_s}\left(\frac{2\gamma\delta(\omega-\omega')\bar{n}_T + \frac{2\kappa\lambda^2_{\text{eff}}}{\tilde{\Delta}^2_{c,\text{eff}}+\kappa^2}\frac{\omega_0}{\omega_s}\delta(\omega-\omega')}{|i\omega_s + \gamma - i\omega|^2}\right. \\
&\left. + \frac{2\gamma\delta(\omega-\omega')(1+\bar{n}_T) + \frac{2\kappa\lambda^2_{\text{eff}}}{\tilde{\Delta}^2_{c,\text{eff}}+\kappa^2}\frac{\omega_0}{\omega_s}\delta(\omega-\omega')}{|i\omega_s + \gamma + i\omega|^2}\right)
\end{aligned} \tag{49}$$

We set $\langle\hat{d}^\dagger(\omega)\hat{d}(\omega')\rangle = 0$, as the photonic polariton mode occupation is vanishingly small. In the last line of equation (49), the first term in the sum corresponds to the blue-shifted sideband, while the second term corresponds to the red-shifted sideband. The asymmetry in the amplitude of the sidebands is thus given by the different factors $\bar{n}_T$ and $(1+\bar{n}_T)$. The expectation value $\langle\delta\hat{a}^\dagger(\omega)\delta\hat{a}(\omega')\rangle$ corresponds to the experimentally observed power spectral density $PSD(\omega)$, excluding the coherent part at $\omega = 0$.

Sideband asymmetry

We can now explicitly calculate the sideband asymmetry in the cavity output photon spectrum, defined as the difference between the spectral weight of the red-shifted and the blue-shifted sideband. With the definition of the mean intracavity photon number in steady state [7]

$$\langle\hat{a}^\dagger\hat{a}\rangle = \frac{1}{2\pi}\int_{-\infty}^{\infty}\int_{-\infty}^{\infty}\langle\hat{a}^\dagger(\omega)\hat{a}(\omega')\rangle\mathrm{d}\omega\mathrm{d}\omega' \tag{50}$$

we call the integral over the blue-shifted sideband $\langle\delta\hat{a}^\dagger\delta\hat{a}\rangle_+$, and the integral over the red-shifted sideband $\langle\delta\hat{a}^\dagger\delta\hat{a}\rangle_-$. The difference between the two sidebands, written as a rate of photons leaving the cavity is thus

$$
\begin{aligned}
&2\kappa\left(\langle\delta\hat{a}^\dagger\delta\hat{a}\rangle_- - \langle\delta\hat{a}^\dagger\delta\hat{a}\rangle_+\right) = \\
&\frac{2\kappa}{2\pi}\int_{-\infty}^{\infty} d\omega \frac{\lambda_{\text{eff}}^2}{\tilde{\Delta}_{c,\text{eff}}^2+\kappa^2}\frac{\omega_0}{\omega_s}\frac{1}{(\omega_s+\omega)^2+\gamma^2}\left(2\gamma(\bar{n}_T+1)+\frac{2\kappa\lambda_{\text{eff}}^2}{\tilde{\Delta}_{c,\text{eff}}^2+\kappa^2}\frac{\omega_0}{\omega_s}\right) - \\
&-\frac{2\kappa}{2\pi}\int_{-\infty}^{\infty} d\omega \frac{\lambda_{\text{eff}}^2}{\tilde{\Delta}_{c,\text{eff}}^2+\kappa^2}\frac{\omega_0}{\omega_s}\frac{1}{(\omega_s-\omega)^2+\gamma^2}\left(2\gamma\bar{n}_T+\frac{2\kappa\lambda_{\text{eff}}^2}{\tilde{\Delta}_{c,\text{eff}}^2+\kappa^2}\frac{\omega_0}{\omega_s}\right) \\
&= \frac{2\kappa}{2\pi}\int_{-\infty}^{\infty} d\omega \frac{\lambda_{\text{eff}}^2}{\tilde{\Delta}_{c,\text{eff}}^2+\kappa^2}\frac{\omega_0}{\omega_s}\frac{2\gamma}{(\omega_s+\omega)^2+\gamma^2} \\
&= 2\kappa\frac{\omega_0}{\omega_s}\frac{\lambda_{\text{eff}}^2}{\tilde{\Delta}_{c,\text{eff}}^2+\kappa^2}
\end{aligned}
\tag{51}
$$

Comparing this result with equation (46), we find a direct relation between the sideband asymmetry and the expectation value for the number of quasi-particles:

$$
2\kappa\left(\langle\delta\hat{a}^\dagger\delta\hat{a}\rangle_- - \langle\delta\hat{a}^\dagger\delta\hat{a}\rangle_+\right) = 2\gamma\left(\langle\hat{c}^\dagger\hat{c}\rangle - \bar{n}_T\right) . \tag{52}
$$

This equation can be interpreted as a rate equation: quasi-particles can be created either via thermal excitation at rate $2\gamma\bar{n}_T$ or via the loss of a red-shifted photon out of the cavity at rate $2\kappa\langle\delta\hat{a}^\dagger\delta\hat{a}\rangle_-$. On the other hand, quasi-particles can be annihilated via the loss of a blue-shifted cavity-photon at rate $2\kappa\langle\delta\hat{a}^\dagger\delta\hat{a}\rangle_+$, or via damping of the c-mode at rate $2\gamma\langle\hat{c}^\dagger\hat{c}\rangle$. These different processes are illustrated in Supplementary Figure 1 together with an energy diagram of the coupled system.

Supplementary Note 2: Data evaluation

The dynamic structure factor

For the Fourier analysis, the detected signal is cut into subtraces of length $0.02\,\mathrm{P}/\mathrm{P}_{\mathrm{cr}}$ corresponding to time windows of 11 ms. For averaging purposes, each subtrace overlaps halfway with the neighboring ones. The two digitized heterodyne quadratures Q_1 and Q_2

are first electronically demodulated at 50 kHz. For each subtrace, we compute the Fourier transform of $Q_1 + iQ_2$ using frequency bins of 90 Hz. We normalize the Fourier transforms to the number of data points in the subtrace and the frequency resolution, and average over all 147 experimental runs. The obtained spectral density $\mathcal{S}(\omega)$ of the intracavity photons is converted into a power spectral density of the light field leaking out of the cavity via $PSD = 10\log\left(\mathcal{S}(\omega)\cdot hc/\lambda_\mathrm{c}\cdot 2\kappa/1\mathrm{mW}\right)$ with the cavity decay rate $\kappa = 2\pi\cdot 1.25\,\mathrm{MHz}$ and the energy per cavity photon of hc/λ_c.

Sideband fitting procedure

A symmetric low-frequency noise feature develops around the strong coherent field in the self-organized phase, which we attribute to technical phase noise between the signal and the LO beam. Sufficiently deep in the self-organized phase, its amplitude is proportional to the coherent field intensity. Before fitting, the scaled averaged technical noise is subtracted from the data in a region up to $\pm 2\,\mathrm{kHz}$. Then, the sidebands appearing in the dynamic structure factor in Fig. 2 are fitted with two damped Lorentzian functions according to Eq. 49: $S_{incoh}(\omega) = BG + \frac{A_+}{((\omega+\omega_s)^2+\gamma^2)^2} + \frac{A_-}{((\omega-\omega_s)^2+\gamma^2)^2}$, where BG is the heterodyne noise level with the signal path blocked, and $A_{+/-}$ the sideband amplitudes.

The imperfect modeling of the technical noise around the phase transition does not permit to start the fit of the sidebands at zero frequency. In order to be dominated by the sideband amplitude and not by noise, we thus restrict the sideband fitting routine to a finite frequency $\nu_{\mathrm{min,fit}}$. This introduces a minimum excitation frequency that can be obtained through the fit. This effect can be observed in Fig. 4 for the quasi-particle frequency very close to the critical point: it is limited to 1 kHz. To account for the influence of the cut-off frequency $\nu_{\mathrm{min,fit}}$, we extract the fit results for $\nu_{\mathrm{min,fit}}$ from 810 Hz (900 Hz in the superradiant phase) to 1170 Hz in 90 Hz steps. Their mean is used as the output of the fitting routine. To determine the error on the fit parameters we select the maximum of one of the three following error estimates: the mean individual fit error, the standard deviation of the fit results for the set of $\nu_{\mathrm{min,fit}}$, or the standard deviation of the fit results for keeping versus not keeping the quasi-particle frequency fixed to the value of the *ab-initio* theory.

Supplementary Note 3: Comparison of *ab-initio* theory with experimental data

The *ab-initio* calculations used in Fig. 1-5 are based on Hamiltonian Eq. (11) with no free fit parameter [3]. They include the two degenerate cavity TEM_{00} modes with circular polarizations ϵ_1 and ϵ_2 that are driven off-resonantly by the transverse laser beam, which is linearly polarized along the y-axis. For the atoms, prepared in the hyperfine state $(F, m_F) = (1, -1)$ with respect to a quantization axis pointing along the cavity axis (x-axis), the ratio of the corresponding two-photon Rabi frequencies is $\eta_1/\eta_2 = 2.66/0.82$. Here, we denoted F as the total angular momentum and m_F the magnetic quantum number. A single maximally coupled atom induces a maximum dispersive shift of the two cavity modes of $U_0^1 = 2\pi \times 71\,\mathrm{Hz}$ and $U_0^2 = 2\pi \times 22\,\mathrm{Hz}$.

We furthermore take into account the measured coherent intracavity photon number and the transverse pump potential that creates a lattice depth of $2.8E_\mathrm{r}$ at the critical point, calibrated using Raman-Nath diffraction [8]. Systematic uncertainties of these quantities are estimated to be 5% each (see shaded regions in Fig. 4 and 5). The Gaussian envelopes of the pump and cavity fields along the transverse directions [9] are effectively included by weighted averages of V_p, $\eta_{1,2}$ and $U_0^{1,2}$ over the spatial extent of the atomic cloud. Here, the Thomas-Fermi radii of the condensate in the external harmonic trapping potential is calculated in the presence of the changing transverse pump lattice and atom number, and is given by $(R_x, R_y, R_z) = (3.5, 8.3, 6.9)$ µm at the critical pump strength, assuming an atom number of $N = 1.0 \times 10^5$.

We use our theoretical model to calculate the dynamic structure factor in Fig. 2 from the PSD using Eq. (1) in the main text with a conversion error of 5%, and to calculate the energy ω_0 of the uncoupled system. The relative coupling strength $\lambda/\lambda_\mathrm{cr}$ shown in the inset of Fig. 3 is calculated from the experimentally determined quasi-particle frequency ω_s and the theoretically obtained frequency of the uncoupled system, ω_0, using $\lambda^2/\lambda_\mathrm{cr}^2 = 1 - \omega_s^2/\omega_0^2$. The energy of the quasi-particle mode shown in Fig. 4 is evaluated using Eq. (38). The theoretical number of quasi-particles in Fig. 5 is calculated using Eq. (51) and (52). The thermal occupancy of the quasi-particle mode shown is evaluated using $\bar{n}_T = \left(\exp[\frac{\hbar\omega_s}{k_B T}] - 1\right)^{-1}$, where k_B is the Boltzmann constant, and $T = 38\,\mathrm{nK}$. The atomic damping rate γ is fitted with a phenomenological function based on a dispersion function: $\gamma(x) = p_0(x-1)/(p_1^2 + (x-1)^2)^{p_2}$. The fit results in the normal phase are $(p_0, p_1, p_2) = (-2\pi \cdot 33.8\,\mathrm{Hz}, 0.039, 1.02)$

and in the self-organized phase, $(p_0, p_1, p_2) = (2\pi \cdot 299\,\mathrm{Hz}, 0.016, 0.649)$. This function is used in Fig. 5 to evaluate the theoretical expectation for the quasi-particle number (grey shaded area).

SUPPLEMENTARY REFERENCES

[1] Hove, L. V. Correlations in Space and Time and Born Approximation Scattering in Systems of Interacting Particles. *Physical Review* **95**(1), 249–262 (1954).

[2] Brennecke, F., Mottl, R., Baumann, K., Landig, R., Donner, T., and Esslinger, T. Real-time observation of fluctuations at the driven-dissipative Dicke phase transition. *Proceedings of the National Academy of Sciences of the United States of America* **110**(29), 11763–11767, July (2013).

[3] Mottl, R., Brennecke, F., Baumann, K., Landig, R., Donner, T., and Esslinger, T. Roton-type mode softening in a quantum gas with cavity-mediated long-range interactions. *Science* **336**, 1570–1573, June (2012).

[4] Nagy, D., Szirmai, G., and Domokos, P. Self-organization of a Bose-Einstein condensate in an optical cavity. *The European Physical Journal D* **48**(1), 127–137, April (2008).

[5] Krämer, M., Menotti, C., Pitaevskii, L., and Stringari, S. Bose-Einstein condensates in 1D optical lattices. *The European Physical Journal D - Atomic, Molecular and Optical Physics* **27**(3), 247–261, December (2003).

[6] Nagy, D., Szirmai, G., and Domokos, P. Critical exponent of a quantum-noise-driven phase transition: The open-system Dicke model. *Physical Review A* **84**(4), 043637, October (2011).

[7] Dimer, F., Estienne, B., Parkins, A., and Carmichael, H. Proposed realization of the Dicke-model quantum phase transition in an optical cavity QED system. *Physical Review A* **75**(1), 013804, January (2007).

[8] Morsch, O. and Oberthaler, M. Dynamics of Bose-Einstein condensates in optical lattices. *Reviews of Modern Physics* **78**(1), 179–215, February (2006).

[9] Baumann, K., Guerlin, C., Brennecke, F., and Esslinger, T. Dicke quantum phase transition with a superfluid gas in an optical cavity. *Nature* **464**, 1301–1306, April (2010).
